# Lack of association between food insecurity, eating disorders, and orthorexia nervosa: findings from a cross-sectional study in Lebanon

**DOI:** 10.3389/fpubh.2025.1572654

**Published:** 2025-09-24

**Authors:** Hala Sacre, Chadia Haddad, Rana Rizk, Joanne Karam, Pascale Salameh

**Affiliations:** ^1^Institut National de Santé Publique, d’Épidémiologie Clinique et de Toxicologie-Liban (INSPECT-LB), Beirut, Lebanon; ^2^Faculty of Public Health, Lebanese University, Fanar, Lebanon; ^3^Research Department, Psychiatric Hospital of the Cross, Jal El Dib, Lebanon; ^4^Department of Nutrition and Food Science, School of Arts and Sciences, Lebanese American University, Byblos, Lebanon; ^5^Gilbert and Rose-Marie Chagoury School of Medicine, Lebanese American University, Byblos, Lebanon; ^6^Faculty of Pharmacy, Lebanese University, Hadat, Lebanon; ^7^Department of Primary Care and Population Health, University of Nicosia Medical School, Nicosia, Cyprus

**Keywords:** food insecurity, eating disorder, orthorexia nervosa, university student, Lebanon

## Abstract

**Objectives:**

To investigate the association between food insecurity (FI), eating disorders (EDs), and orthorexia nervosa (ON) among Lebanese university students.

**Methods:**

This cross-sectional study, conducted between 2021 and 2022, enrolled 197 students, from various majors, via snowball sampling. Data were collected on Google Forms via social media platforms.

**Results:**

Most participants (59.4%) declared being food-insecure, without financial support (67.0%), nor financial independence (68.5%). Most did not show any ED (81.7%) or a risk of ON (79.7%). Reported EDs were bulimia-nervosa (6.1%) and anorexia-nervosa (6.1%); 4.1% had a high risk of ON. No significant associations were found between declared FI, ED, and the risk of ON. Higher adherence to the Mediterranean diet (ORa = 1.31) and higher perceived stress (ORa = 1.14) were significantly associated with EDs. Higher exercise addiction scores (ORa = 1.25) and higher insomnia levels (ORa = 1.26) were significantly associated with the risk of ON. Being employed (ORa = 0.17) and skipping meals (ORa = 0.20) were inversely associated with declared ON.

**Conclusion:**

FI and EDs were not associated among university students in Lebanon. Research into underlying mechanisms and cultural aspects is crucial to clarifying these associations.

## Introduction

Food insecurity (FI), defined “limited or uncertain availability of nutritionally adequate and safe food or limited or uncertain ability to acquire acceptable foods in socially acceptable ways,” due to a lack of money or other resources, is a global public health problem (1–3). It is especially concerning among vulnerable populations such as university students, given their financial constraints and limited access to nutritious foods (4). This multifaceted phenomenon has deleterious consequences for university students, extending beyond poor dietary outcomes (5) and nutritional deficiencies to disrupt physical and mental health, academic outcomes (6), and well-being. In this population, FI is estimated to reach up to 42% (6); these figures were exacerbated after the COVID-19 pandemic (7).

Eating disorders (EDs) are common in university settings (8, 9) and are characterized by abnormal eating habits and negative body image; frequent forms include anorexia nervosa (AN), bulimia nervosa (BN), and binge-eating disorder (BED) (10). The association between FI and EDs appears bidirectional (11) and persists after accounting for depression and anxiety, suggesting specific mechanisms beyond general psychological distress (12). Cycles of scarcity followed by re-feeding may heighten hunger and overeating, reinforcing compensatory behaviors (13). Conversely, the financial and behavioral demands of disordered eating, such as extensive physical activity or acquiring specific dietary items, may increase vulnerability to FI (11). Evidence among university students (11, 14–17) suggests that FI is associated with disordered-eating pathology and positive ED screens (13, 16), especially bulimic-spectrum EDs (11), binge eating, and compensatory fasting (13).

Orthorexia nervosa (ON) —a maladaptive preoccupation with healthy eating (18)—has been observed in student populations (8), yet its intersection with FI remains unexplored. Most evidence on FI and disordered eating among university students derives from Western contexts (13, 14, 19, 20). In these studies, FI often co-occurs with ED pathology (14, 21). Given the role of culture in shaping what is considered healthy eating and expectations of body image, a closer look into this association in non-Western settings, including Arab countries, is needed (22, 23).

Since 2019, Lebanon has been grappling with a protracted economic and financial crisis (24), compounded by the COVID-19 pandemic and the aftermath of the Beirut port explosion, with nationally high levels of FI, including an estimate of 19% with acute FI an expectations of a worsened condition in the months ahead (25). Among Lebanese university students, FI prevalence has been reported at 43% in 2021 (26) and 59% in 2022 (27), indicating substantial vulnerability within this group. Concurrently, research in Lebanon documents elevated weight preoccupation and strong thin-ideal pressures among students (28). Taken together, these contextual factors warrant exploring the co-existence of FI with EDs in this population group.

Therefore, this study examined whether self-reported FI is associated with EDs and ON among Lebanese university students.

## Methods

### Study design

This online cross-sectional study surveyed 197 Lebanese university students between November 1, 2021, and March 31, 2022, using a questionnaire created on Google Forms. Non-probability sampling was employed to recruit participants, where the survey link was shared on various social media platforms (Facebook, WhatsApp, and Instagram). The questionnaire was developed in English, as all participating university students had the proficiency to comprehend it and respond to the questions and it required 20 to 30 min to be completed. A pilot test was conducted with five university students to assess the questionnaire’s acceptance and clarity. The data from the pilot were added to the final dataset because no changes were made following the pilot testing.

### Sampling and inclusion criteria

Inclusion criteria consisted of being a current university student (undergraduate or postgraduate) aged over 17 years, regardless of the major of study. Participants with a self-reported history of diagnosed depression, food addiction, or restrictive eating behaviors prior to the study were excluded from participation. Participation was voluntary and anonymous, and participants could withdraw from the study at any moment. All participants provided consent by clicking on an “I agree to participate in this survey, proceed” button, available in the questionnaire’s introductory section, before proceeding to the survey. Participants were not remunerated or offered any other benefits for their participation.

### Ethics approval and consent to participate

The study was conducted according to the Declaration of Helsinki. Ethics approval was obtained *a priori* from the INSPECT-LB (Institut National de Santé Publique, d’Épidémiologie Clinique et de Toxicologie-Liban) Research Ethics Committee (2021REC-002-INSPECT-09-17). All participants provided informed consent at the beginning of the survey before they could access the questionnaire.

### Study variables

The survey included measures of FI, financial well-being, EDs, risk of ON, and adherence to a healthy eating pattern, i.e., the Mediterranean diet. It also collected information on physical activity, sleep quality, stress, and sociodemographic characteristics. The following scales were used in the study:

The Household Food Insecurity Access Scale (HFIAS) is an 18-item instrument consisting of two sets of questions, i.e., nine “occurrence” and nine “frequency-of-occurrence” questions. Participants answered all the items based on their household experience of food security over the past 30 days (29). In each instance, respondents were asked about the occurrence of a condition (yes or no), and, in the case of a positive answer, how frequently they experienced it; response options were rarely (score = 1), occasionally (score = 2), or often (score = 3). Responses were then converted into a continuous or categorical food security indicator. The values were then added together to calculate the HFIAS on a continuous scale. The total HFIAS can range from 0 to 27, categorizing households as food-secure, mildly food-insecure, moderately food-insecure, or severely food-insecure. This scale was previously validated in Lebanon, with a Cronbach’s alpha of 0.91 and a moderate agreement in the test–retest reliability (ICC = 0.58) (30). In the present study, the score was dichotomized into food-secure versus food-insecure (including mild, moderate and severe food-insecure) and the Cronbach’s alpha value was 0.858.

The Eating Disorder Diagnostic Scale (EDDS) is a 22-item self-report measure is designed for use in adolescents and adults (13–65 years old). It assesses eating disorders such as BED, BN, and AN by asking about eating habits, body image, and compensatory behaviors over the previous three to six months (31). The EDDS is a combination of Likert, dichotomous, and frequency scores, as well as open-ended questions, including weight and height. It comprises a diagnostic scale that represents participants’ types of eating pathology, which can identify BED, BN, and AN as per the recommendations and syntax provided by the authors of the original research (31). In the present study, the diagnosis scale was further divided into two categories: people with any eating disorder and those with no eating disorder.

The Düsseldorf Orthorexia Scale (DOS) is a self-reported 10-item assessment tool is used to evaluate orthorexic eating behaviors rated on a 4-point Likert scale, ranging from “this applies to me” (4 points) to “this does not apply to me” (1 point). The maximum score is 40 points, with higher values indicating higher levels of orthorexic behavior (32). A score of 30 or above is considered the cut-off point for the presence of ON, while a score between 25 and 29 indicates a risk of ON (33). The DOS was validated in Arabic among Lebanese adolescents, demonstrating good structural validity and internal consistency (Cronbach’s alpha = 0.85) (34). In this study, Cronbach’s alpha was 0.856.

The Revised Exercise Addiction Inventory (EAI-R) comprises six items representing the six components of addictive behaviors, i.e., salience, mood modification, tolerance, withdrawal symptoms, conflict, and relapse (35). Each of the six items is rated on a 6-point scale, ranging from 1 (strongly disagree) to 6 (strongly agree). The exercise addiction risk score is calculated by summing the scores of all the items, where higher scores indicate a higher risk of exercise addiction. In this study, Cronbach’s alpha was 0.844.

The Mediterranean Diet Adherence Screener (MEDAS) is a set of 14 items designed to measure the extent of adherence to the Mediterranean diet. It consists of twelve items about food frequency and two items related to dietary habits. Responses are rated on a dichotomous scale of 0 (no adherence) and 1 (adherence) (36). The total score ranges between 0 and 14, with higher scores indicating greater adherence. While the MEDAS has been widely used in numerous Lebanese studies (37–39), it has not yet been validated in Lebanon. In this study, Cronbach’s alpha was 0.319.

The Pittsburgh Sleep Quality Index (PSQI) is a 19-item self-reported questionnaire that evaluates sleep quality over one month (40). It generates seven components, i.e., subjective sleep quality, sleep latency, sleep duration, habitual sleep efficiency, sleep disturbances, use of sleeping medication, and daytime dysfunction, yielding one global score calculated by summing all responses on 0–3 interval scale. The total score ranges from 0 to 21, with lower scores indicating better sleep quality. The PSQI was validated in Arabic in a sample of healthy Arab Americans, demonstrating acceptable reliability (Cronbach’s alpha = 0.65) (41). In this study, Cronbach’s alpha was 0.836.

The International Physical Activity Questionnaire (IPAQ)-Short Form consists of seven questions about the frequency and duration of light, moderate, and intense physical exercise (42). Questions ask about participants’ daily activity levels, including how much time they spent on each of these activities and if they walked or engaged in moderate-to-vigorous-intense activities for at least ten minutes. The last question is about the time (in hours) spent sitting per weekday over the past seven days. A logarithmic transformation was used to improve the normality of the scale. The Metabolic Equivalent of Tasks (METs) were then calculated by multiplying the total minutes spent doing the activity by the frequency and by the respective constants of 3.3, 4.0, and 8.0 for light, moderate, and vigorous activity. The respective MET values for all activities performed in bouts of more than 10 min are then summed to yield a total MET score. The IPAQ was validated in Lebanon, demonstrating high reliability (Cronbach’s alpha between 0.769 and 1.00), with intraclass correlation coefficients (ICC) ranging from 0.625 to 0.999 and (*p* < 0.001) (43).

The Perceived Stress Scale (PSS-10) is a 10-item tool that measures perceived stress perception, where respondents describe their current life as unpredictable, uncontrollable, and stressful, using options from never (0) to very often (4), with a total score ranging from 0 to 40. Higher scores indicate higher perceived stress (44). This scale was validated in Lebanon and demonstrated good test–retest reliability (Cronbach’s = 0.74) (45). In this study, Cronbach’s alpha was 0.756.

The InCharge Financial Distress/Financial Well-Being (IFDFW) Scale is an 8-item self-reported instrument that evaluates participants’ perceived levels of financial distress/financial well-being on a linear scale from 1 (overwhelming financial distress/lowest level of financial well-being) to 10 (no financial distress/highest level of financial well-being) (46). In this study, Cronbach’s alpha was 0.844.

Factor analysis of the scales used in this study is available in the Supplementary Material.

### Sample size calculation

The minimum sample size was calculated using the G*Power software 3.1.9.7 for Windows (Heinrich Heine, Universität Düsseldorf, Düsseldorf, Germany). As the major dependent variables are quantitative, multiple regressions would be used to assess their correlates. Assuming a calculated effect size is f2 = 0.1 (small effect size) related to the Omnibus test of the multiple regression, the minimum necessary sample will be *n* = 185, considering an alpha error of 5%, a power of 80%, and allowing 12 predictors to be included in the model.

### Statistical analysis

Statistical analysis was conducted using the Statistical Package for the Social Sciences (SPSS) version 25.0. Quantitative data were presented as means and standard deviations, while qualitative data were shown as frequencies and percentages. As the dependent variables (ED and risk of ON) were categorical, the Chi-square test was used in cases where the expected cell count was higher than 5. When the assumption was not met, the Wilcoxon and Fisher’s exact tests were used, respectively. When comparing categorical and quantitative variables, the student’s t-test was used.

Regarding multivariable analysis, two logistic regressions were performed using the ENTER method. Variables that showed a *p* < 0.2 in the bivariate analysis were entered into the regression models. The first regression analysis considered the ED (presence vs. absence) as the dependent variable. The second regression analysis took the risk of ON (presence vs. absence) as the dependent variable. Moreover, the internal consistency of the scales was assessed using Cronbach’s alpha. A *p*-value less than 0.05 was considered significant.

## Results

### Description of the sample and eating disorders

Table 1 presents the sociodemographic and other characteristics of the 197 university students who participated in the study. The majority of participants experienced FI (59.4%), were unemployed (62.4%), and lived in an urban area (66.0%). Most were single (85.3%), female (80.2%), had low or no income (63.5%), relied on their parents as the source of income (62.9%), and resided off campus with parents or guardians (89.3%). Also, the majority had no financial support (67.0%) and were not financially independent (68.5%). The mean age of participants was 22.32 ± 5.14 years with a range of 17–48 years, and the mean BMI was 22.91 ± 4.29 kg/m^2^.

**Table 1:** Sociodemographic and other characteristics of the sample (*n* = 197 participants).

Variable	Frequency (%)
(A) Sociodemographic characteristics	
Gender	
Male	39 (19.8%)
Female	158 (80.2%)
Marital status	
Single	168 (85.3%)
Solid partnership/not married	14 (7.1%)
Married	11 (5.6%)
Widowed	1 (0.5%)
Divorced	3 (1.5%)
Financial independence	
Yes	62 (31.5%)
No	135 (68.5%)
Monthly income	
No income	77 (39.1%)
Low	48 (24.4%)
Intermediate	41 (20.8%)
High	31 (15.7%)
Source of income	
Parents or guardians	124 (62.9%)
Parents plus own occupation	28 (14.2%)
Own occupation	45 (22.8%)
Financial support	
Yes	65 (33.0%)
No	132 (67.0%)
Employment status	
Full time employed	39 (19.8%)
Part time/marginally employed	35 (17.8%)
Not employed	123 (62.4%)
Place of residence	
On campus	6 (3.0%)
Off campus, not with parents/guardians	15 (7.6%)
Off campus, with parents/guardians	176 (89.3%)
Region of residence	
In the city or rather urban	130 (66.0%)
In the country side or rather rural	67 (34.0%)
Household food insecurity	
Food secure	80 (40.6%)
Food insecure	117 (59.4%)
	Mean ± SD
Age	22.32 ± 5.14 [Range 17–48]
(B) Health-related and behavioral measures	
BMI	22.91 ± 4.29
MEDAS	5.44 ± 1.89
PSS	21.64 ± 6.19
EAI	17.90 ± 7.07
IPAQ (log10)	3.08 ± 0.54
PSQI	6.83 ± 3.25
IFDFW	40.01 ± 9.27

BMI: Body Mass Index; SD: Standard Deviation; MEDAS: Mediterranean Diet Adherence Screener; PSS: Perceived Stress Scale; EAI: Exercise addiction inventory; IPAQ (log10): International Physical Activity Questionnaires Logarithm to the base 10; PSQI: Pittsburgh Sleep Quality Index; IFDFW: InCharge Financial Distress/Financial Well-Being scale.

**Figure 1:**
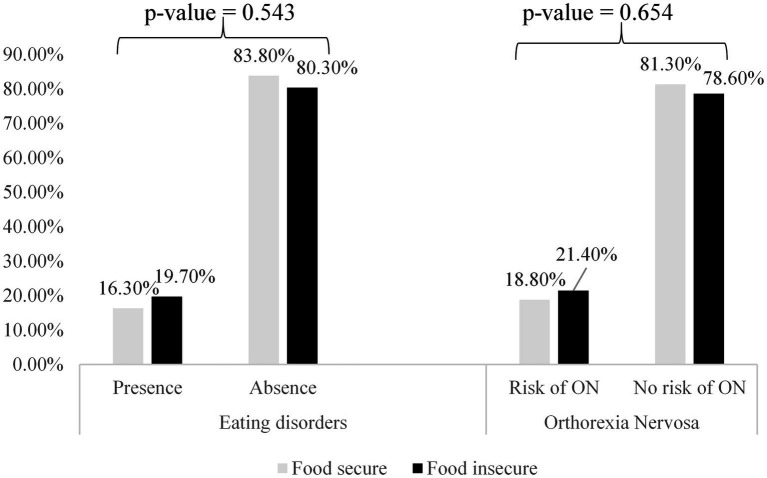
Association between self-reported eating disorder, risk of orthorexia nervosa, and food security (sample size = 197 participants) (food-insecurity and eating-disorders in students, Lebanon, 2021–2022).

As shown in Table 2, most students did not show any ED (81.7%) or a risk of ON (79.7%). The most frequently declared EDs in the sample were BN (6.1%) and AN (6.1%). Only 4.1% of the sample had a high risk of ON. No significant associations were found between FI, ED, and the risk of ON (*p* > 0.05). Among those who exhibited EDs, 16.3% were food secure, compared with 19.7% who were food insecure (*p* = 0.543). Among those with a high risk of ON, 18.8% were food secure, while 21.4% were food insecure (*p* = 0.654) (Figure 1).

**Table 2:** Description of self-reported eating disorders (sample size = 197 participants) (food-insecurity and eating-disorders in students, Lebanon, 2021–2022).

Self-reported eating disorders	Frequency (%)
Eating disorder	
ED	161 (81.7%)
BN	12 (6.1%)
BED	2 (1.0%)
Atypical anorexia nervosa	12 (6.1%)
Low frequency BN	1 (0.5%)
Low frequency BED	1 (0.5%)
Night eating syndrome	8 (4.1%)
Risk of ON, as measured by the Düsseldorf Orthorexia scale	
No risk	157 (79.7%)
Moderate risk	32 (16.2%)
High risk	8 (4.1%)

ED: Eating Disorder; BN: Bulimia Nervosa; BED: Binge Eating Disorder; ON: Orthorexia Nervosa.

### Bivariate analysis

Table 3 presents the bivariate analysis, with self-reported EDs and the risk of ON as the respective dependent variables. The analysis showed that FI tended to be higher among participants with EDs and at risk of ON; however, these differences did not reach statistical significance (*p* > 0.05). When considering EDs as the dependent variable, participants with EDs had significantly higher mean MEDAS scores (6.10 vs. 5.31, *p* = 0.034) and higher mean PSS scores (25.36 vs. 20.81, *p* < 0.001) compared with those without EDs. When considering the risk of ON as the dependent variable, a significantly higher proportion of participants with a risk of ON did not skip meals compared with those who skipped meals (29.9% vs. 12.7%, *p* = 0.003).

**Table 3:** Bivariate analysis taking the eating disorders and the risk of orthorexia nervosa as the dependent variables (food-insecurity and eating-disorders in students, Lebanon, 2021–2022).

Variable	Eating disorders		Orthorexia nervosa	
	Presence [36 (18.3%)]	Absence [161 (81.7%)]	Risk of ON [40 (20.3%)]	No risk of ON [157 (79.7%)]
Gender				
Male	7 (17.9%)	32 (82.1%)	7 (17.9%)	32 (82.1%)
Female	29 (18.4%)	129 (81.6%)	33 (20.9%)	125 (79.1%)
*p*-value	0.953		0.683	
Marital status				
Single/widowed/divorced	32 (18.6%)	140 (81.4%)	35 (20.3%)	137 (79.7%)
Married/ solid relationship	4 (16.0%)	21 (84.0%)	5 (20.0%)	20 (80.0%)
*p*-value	0.753		0.968	
Financial independence				
Yes	15 (24.2%)	47 (75.8%)	11 (17.7%)	51 (82.3%)
No	21 (15.6%)	114 (84.4%)	29 (21.5%)	106 (78.5%)
*p*-value	0.145		0.545	
Monthly income				
No income	8 (10.4%)	69 (89.6%)	17 (22.1%)	60 (77.9%)
Low	9 (18.8%)	39 (81.3%)	10 (20.8%)	38 (79.2%)
Intermediate	9 (22.0%)	32 (78.0%)	7 (17.1%)	34 (82.9%)
High	10 (32.3%)	21 (67.7%)	6 (19.4%)	25 (80.6%)
*p*-value	0.054		0.932	
Source of income				
Parents or guardians	18 (14.5%)	106 (85.5%)	29 (23.4%)	95 (76.6%)
Parents plus own occupation	7 (25.0%)	21 (75.0%)	5 (17.9%)	23 (82.1%)
Own occupation	11 (24.4%)	34 (75.6%)	6 (13.3%)	39 (86.7%)
*p*-value	0.205		0.336	
Financial support				
Yes	14 (21.5%)	51 (78.5%)	17 (26.2%)	48 (73.8%)
No	22 (16.7%)	110 (83.3%)	23 (17.4%)	109 (82.6%)
*p*-value	0.405		0.152	
Employment status				
Employed	17 (23.0%)	57 (77.0%)	10 (13.5%)	64 (86.5%)
Not employed	19 (15.4%)	104 (84.6%)	30 (24.4%)	93 (75.6%)
*p*-value	0.186		0.066	
Place of residence				
On campus	1 (16.7%)	5 (83.3%)	1 (16.7%)	5 (83.3%)
Off campus	35 (18.3%)	156 (81.7%)	39 (20.4%)	152 (79.6%)
*p*-value	0.918		0.822	
Region of residence				
In the city or rather urban	23 (17.7%)	107 (82.3%)	26 (20.0%)	104 (80.0%)
In the country side or rather rural	13 (19.4%)	54 (80.6%)	14 (20.9%)	53 (79.1%)
*p*-value	0.768		0.882	
Skip meals				
Yes	22 (20.0%)	88 (80.0%)	14 (12.7%)	96 (87.3%)
No	14 (16.1%)	73 (83.9%)	26 (29.9%)	61 (70.1%)
*p*-value	0.481		0.003	
Household food insecurity				
Food secure	13 (16.3%)	67 (83.8%)	15 (18.8%)	65 (81.3%)
Food insecure	23 (19.7%)	94 (80.3%)	25 (21.4%)	92 (78.6%)
*p*-value	0.543		0.654	
	Mean ±SD	Mean ±SD	Mean ±SD	Mean ±SD
Age	22.89 ± 4.83	22.20 ± 5.21	21.50 ± 3.75	22.54 ± 5.42
*p*-value	0.468		0.257	
MEDAS	6.10 ± 2.00	5.31 ± 1.85	6.39 ± 1.88	5.21 ± 1.83
*p*-value	0.034		0.001	
PSS	25.36 ± 6.57	20.81 ± 5.81	22.37 ± 6.08	21.46 ± 6.23
*p*-value	<0.001		0.409	
EAI	18.36 ± 8.18	17.80 ± 6.82	24.47 ± 5.98	16.23 ± 6.32
*p*-value	0.672		<0.001	
IPAQ (log10)	3.13 ± 0.45	3.07 ± 0.56	3.25 ± 0.45	3.03 ± 0.56
*p*-value	0.577		0.029	
PSQI	7.65 ± 2.90	6.66 ± 3.31	7.68 ± 6.91	6.63 ± 3.05
*p*-value	0.109		0.127	
IFDFW	38.13 ± 9.02	40.43 ± 9.31	39.70 ± 9.64	40.09 ± 9.21
*p*-value	0.180		0.811	

The statistical tests used in this table were as follows: the Chi-square test was applied to examine the relationship between two categorical variables, while the Student’s t-test was used to compare categorical and quantitative variables. Values marked in bold are significant. SD: Standard Deviation; MEDAS: Mediterranean Diet Adherence Screener; PSS: Perceived Stress Scale; EAI: Exercise addiction inventory; IPAQ (log10): International Physical Activity Questionnaires Logarithm to the base 10; PSQI: Pittsburgh Sleep Quality Index; IFDFW: InCharge Financial Distress/Financial Well-Being scale.

Also, participants with a risk of ON had significantly higher mean MEDAS scores (6.39 vs. 5.21, *p* = 0.001), higher mean EAI scores (24.47 vs. 16.23, *p* < 0.001), and higher mean log10 physical activity levels (3.25 vs. 3.03, *p* = 0.029) compared with those without a risk of ON. The remaining variables were not associated with EDs or the risk of ON (*p* > 0.05 for all).

### Multivariable analysis

No significant association was found between self-reported FI and EDs or ON (*p* > 0.05) in the multivariable analysis. In the first logistic regression model, with EDs (presence vs. absence) as the dependent variable, higher MEDAS (Adjusted Odds Ratio, ORa = 1.31), and higher PSS (ORa = 1.14) scores were significantly associated with the presence of EDs (Table 4, Model 1).

**Table 4:** Multivariable analysis (food-insecurity and eating-disorders in students, Lebanon, 2021–2022).

Variable	ORa	*p*-value	95% confidence interval	
Lower bound	Upper bound	
Model 1: Logistic regression with EDs (presence vs. absence*) as the dependent variable				
HFIAS	1.098	0.848	0.421	2.862
Financial independence (no vs. yes*)	1.385	0.651	0.338	5.677
Income level (low vs. no income*)	1.840	0.354	0.506	6.687
Income level (intermediate vs. no income*)	3.713	0.059	0.953	14.470
Income level (high vs. no income*)	3.802	0.082	0.845	17.098
Employment status (employed vs. not employed*)	1.017	0.982	0.251	4.117
MEDAS	1.311	0.028	1.029	1.669
PSS	1.145	0.001	1.056	1.242
PSQI	0.986	0.851	0.855	1.138
IFDFW	0.995	0.856	0.942	1.051
Model 2: Logistic regression with the risk of ON (presence vs. absence*) as the dependent variable				
HFIAS	1.887	0.271	0.609	5.848
Financial support (no vs. yes*)	0.872	0.792	0.313	2.425
Employment status (employed vs. not employed*)	0.178	0.006	0.053	0.606
Skipping meals (yes vs. no*)	0.207	0.004	0.070	0.612
MEDAS	1.174	0.217	0.910	1.515
EAI-R	1.254	0.000	1.135	1.384
IPAQ (log10)	1.547	0.448	0.501	4.775
PSQI	1.265	0.007	1.066	1.502

*Reference group. Ora: Adjusted Odds Ratio; EDs: Eating Disorders; HFIAS: Household Food Insecurity Access Scale; MEDAS: Mediterranean Diet Adherence Screener; PSS: Perceived Stress Scale; PSQI: Pittsburgh Sleep Quality Index; IFDFW: InCharge Financial Distress/Financial Well-Being scale; EAI-R: Revised exercise addiction inventory; IPAQ (log10): International Physical Activity Questionnaires Logarithm to the base 10.

In the second logistic regression model, with the risk of ON (presence vs. absence) as the dependent variable, higher exercise addiction scores (ORa = 1.25) and higher insomnia levels (ORa = 1.26) were significantly associated with the risk of ON. However, being employed (ORa = 0.17) and skipping meals (ORa = 0.20) were inversely associated with declared ON (Table 4, Model 2).

## Discussion

In this cross-sectional sample of Lebanese university students, no association was found between self-reported FI and either EDs or ON after adjustment for potential confounding factors. Most participants reported FI, yet the majority screened negative for EDs and ON risk. Greater adherence to a Mediterranean diet and higher perceived stress were associated with EDs, while higher exercise addiction and insomnia scores were associated with ON risk; employment and meal-skipping were inversely related to ON.

FI prevalence in this cohort was high (≈60%), exceeding estimates reported in several high-income and upper-middle-income contexts, such as Germany (33.7%) (27), the United States (29%) (47), or Turkey (35.4%) (48), surpassing pre-crisis Lebanese estimates of 39–43% (26, 49) while aligning with the 59% reported in Lebanon in 2022 (27). This pattern is consistent with crisis-related deterioration flagged by the 2023 IPC report (25) and with indicators of financial strain among students, e.g., limited financial support (67.0%) and independence (68.5%). FI has been linked to stress, anxiety, and emotional distress, affecting academic performance (50). Food-insecure students often prioritize work over studies (51) and affordable over nutritional and quality foods (52, 53), highlighting the need for integrated financial and food-security support.

Despite the high prevalence of FI (59.4%), clinically significant EDs were uncommon (81.7%), and high ON risk was rare (79.7%). The most prevalent eating disorders were BN and AN (each 6.1%) were, with a low percentage of students being at high risk of ON (4.1%). FI was not associated with the presence or risk of ED or ON (*p* > 0.05) in the present study. These findings differ from reports linking FI to eating-disorder pathology in student and adult samples (11, 12, 14, 54). This discrepancy may reflect the timing and nature of FI tied to Lebanon’s recent economic crisis and broader socioeconomic factors, warranting further research into these pathways and their implications for mental health and eating behaviors.

Higher levels of exercise addiction and insomnia were significantly associated with an increased likelihood of ON. Previous research had described the potential link between ON and excessive exercise behaviors (55). This association aligns with the notion that individuals with ON may have a fixation on maintaining a perceived healthy lifestyle, which could manifest in excessive exercise routines. However, the relationship between ON and sleep disturbances is less clear, with conflicting findings in the literature. While some studies support our results (56), others have reported opposing tendencies regarding sleep patterns and ON (57). Further research is necessary to examine the various factors involved and clarify these associations.

Interestingly, being employed and skipping meals were inversely associated with ON. One potential explanation is the protective effect of employment due to the structured routine and social interactions at work, which may counterbalance the tendency towards obsessive dietary behaviors. The inverse association with skipping meals seems counterintuitive, as disordered eating patterns are often associated with ON (58). However, this finding may suggest that individuals with ON are less likely to intentionally skip meals due to their fixation on adhering to strict dietary rules and patterns.

Our results add to the controversy surrounding the complex relationship between FI and EDs among university students. Further research is required to confirm our findings and elucidate the underlying mechanisms and potential confounding factors contributing to these associations.

### Strengths and limitations

This study contributes to the existing body of evidence on the potential relationship between FI and EDs among university students in an economic crisis context; it also pioneers exploring the association between FI and ON. It employed validated scales to assess self-reported EDs, physical activity, diet, and FI, adding strengths to the findings.

However, it has some limitations that should be acknowledged. The study was conducted on a volunteer sample of university students, introducing the potential for selection bias and the limited generalizability of the results to the broader Lebanese population. The possibility of residual confounding despite multivariable analysis and recall bias as a source of measurement error cannot be ruled out. Future research should aim for a more representative sample, with a higher proportion of male participants, and consider using more rigorous techniques, such as clinical assessments for eating disorders, food records for nutritional evaluation, objective measures of physical activity and energy expenditure, and sleep diaries, to accurately capture the different concepts since self-reported measures could always be subject to information bias; note that the latter is expected to be non-differential, and would drive the results towards the null hypothesis.

Addressing these limitations could further confirm our findings, show additional association and causality relationships, and refine our understanding of the complex interplay between eating behaviors, food insecurity, and the unique challenges students face in times of economic crisis.

## Conclusion

This study did not find a relationship between self-reported food insecurity and eating disorders among university students in Lebanon amid significant socioeconomic challenges. While declared FI was highly prevalent, its association with ED and ON was not significant. Factors like perceived stress, Mediterranean diet adherence, exercise addiction, insomnia, employment, and skipping meals showed intricate links with EDs and ON. Further research into underlying mechanisms, confounding factors, and cultural aspects is crucial to clarifying these associations, while tailored interventions addressing financial instability, mental health, and balanced lifestyle practices could improve students’ well-being.

## Data Availability

The raw data supporting the conclusions of this article will be made available by the authors, without undue reservation.

## Glossary

AN: Anorexia Nervosa
BED: Binge-Eating Disorder
BN: Bulimia Nervosa
DOS: The Düsseldorf Orthorexia Scale
EAI-R: The Revised Exercise Addiction Inventory
EDDS: The Eating Disorder Diagnostic Scale
EDs: Eating Disorders
FI: Food Insecurity
HFIAS: The Household Food Insecurity Access Scale
ICC: Intraclass Correlation Coefficients
IFDFW: The InCharge Financial Distress/Financial Well-Being
INSPECT-LB: Institut National de Santé Publique d’Épidémiologie Clinique et de Toxicologie-Liban
IPAQ: The International Physical Activity Questionnaire
MEDAS: The Mediterranean Diet Adherence Screener
METs: Metabolic Equivalent of Tasks
ON: Orthorexia Nervosa
ORa: Adjusted Odds Ratio
PSQI: The Pittsburgh Sleep Quality Index
PSS-10: The Perceived Stress Scale
SPSS: Statistical Package for the Social Sciences
