# Supplementary material for: Lack of association between food insecurity, eating disorders, and orthorexia nervosa: findings from a cross-sectional study in Lebanon
Source: Front Public Health. 2025 Sep 24;13:1572654. doi: 10.3389/fpubh.2025.1572654 (PMC12504193; doi:10.3389/fpubh.2025.1572654)
Supplement: Supplementary file 1 [file Table_1.DOCX]

**Supplementary Material: Factor analysis of the scales used in this study**

**Table S1: Factor analysis of the Düsseldorf Orthorexia Scale (Food-insecurity and eating-disorders in students, Lebanon, 2021-2022).**

| **Promax rotated matrix** | | |
| --- | --- | --- |
| **Factor** | **Factor 1** | **Factor 2** |
| DOS: 5. I think it is positive to pay more attention to healthy eating than other people. | .857 |  |
| DOS: 1. That I eat healthy food is more important to me than pleasure. | .805 |  |
| DOS: 2. I have established dietary rules. | .803 |  |
| DOS: 3. I can enjoy food/food only if I am sure it is healthy. | .733 |  |
| DOS: 8. My thoughts constantly revolve around healthy eating and I adjust my daily routine accordingly. | .624 |  |
| DOS: 10. When I have eaten something unhealthy, I feel down. |  | .921 |
| DOS: 6. If I eat something unhealthy, I blame myself a lot. |  | .879 |
| DOS: 7. I feel that I am ostracized by friends and colleagues because of my strict dietary standards. |  | .739 |
| DOS: 9. It is difficult for me to go against my dietary rules. |  | .507 |
| DOS: 4. I try to avoid an invitation to dinner at a friend's house if they are not careful about healthy eating. |  | .355 |
| **Percentage variance explained = 58.90%** | 44.18 | 14.72 |
| **Kaiser-Meyer-Olkin (KMO)** | 0.844 | |
| **Bartlett’s test of sphericity** | p < 0.001 | |

DOS: Düsseldorf Orthorexia Scale; KMO: Kaiser-Meyer-Olkin

**Table S2: Factor analysis of the Perceived Stress Scale (10 item version) (Food-insecurity and eating-disorders in students, Lebanon, 2021-2022).**

| **Promax rotated matrix** | | |
| --- | --- | --- |
| **Factor** | **Factor 1** | **Factor 2** |
| PSS3: In the past month, how often have you felt nervous or 'stressed'? | .842 |  |
| PSS1. In the last month, how often were you surprised by unexpected events? | .840 |  |
| PSS2: In the past month, how often have you felt unable to control important things in your life? | .814 |  |
| PSS10: How often did you feel that difficulties were piling up so much that they were over your head? | .802 |  |
| PSS9: How often did you feel angry about things that were out of your control? | .785 |  |
| PSS6: In the past month, how often did you feel that you were unable to cope with tasks that came up? | .763 |  |
| PSS5: In the past month, how often did you feel that things in your life were going exactly as they should? |  | .826 |
| PSS8: How often did you feel in control of the situation? |  | .795 |
| PSS4: In the past month, how often have you felt confident that you are able to handle personal problems? |  | .789 |
| PSS7: How often have you been able to deal with life's adversities in a controlled manner? |  | .784 |
| **Percentage variance explained = 65.55%** | 39.98 | 25.56 |
| **Kaiser-Meyer-Olkin (KMO)** | 0.847 | |
| **Bartlett’s test of sphericity** | p < 0.001 | |

PSS: Perceived Stress Scale; KMO: Kaiser-Meyer-Olkin

**Table S3: Factor analysis of the Revised Exercise Addiction Inventory (Food-insecurity and eating-disorders in students, Lebanon, 2021-2022).**

| **Promax rotated matrix** | |
| --- | --- |
| **Factor** | **Factor 1** |
| EAI-R 6: If I reduce the amount of my workout and then start again, I always end up back at the amount I did before. | .826 |
| EAI-R 3: I use exercise as a way to change my mood (e.g., to get pumped up, to vent). | .822 |
| EAI-R 4: Over time, I have increased the amount of exercise I do per day. | .821 |
| EAI-R 5: When I have to skip a workout, I feel cranky and irritable. | .808 |
| EAI-R 1: Exercise is the most important thing in my life. | .804 |
| EAI-R 2: Conflicts have already arisen between me and my family and/or partner regarding the amount of exercise I do. | .367 |
| **Percentage variance explained** | 57.77 |
| **Kaiser-Meyer-Olkin (KMO)** | 0.847 |
| **Bartlett’s test of sphericity** | p < 0.001 |

EAI-R: Revised Exercise Addiction Inventory; KMO: Kaiser-Meyer-Olkin

**Table S4: Factor analysis of the InCharge Financial Distress/Financial Well-Being Scale (Food-insecurity and eating-disorders in students, Lebanon, 2021-2022).**

| **Promax rotated matrix** | | |
| --- | --- | --- |
| **Factor** | **Factor 1** | **Factor 2** |
| IFDFW 5: How confident are you that you can raise the money to cover a financial emergency, which is about 1000? | .849 |  |
| IFDFW 7: How often do you just get by, living paycheck to paycheck? | .759 |  |
| IFDFW 6: How often does this happen to you? You want to go out to eat, see a movie at the theater, or do something else and you don't go because you can't afford it. | .754 |  |
| IFDFW 4: How often do you worry about meeting your normal monthly living expenses? | .650 |  |
| IFDFW 2: How satisfied are you with your current financial situation? | .460 |  |
| IFDFW 8: How stressed do you feel about your personal finances in general? |  | .965 |
| IFDFW 1: How high do you feel the level of your financial stress is today? |  | .910 |
| IFDFW 3: How do you feel about your current financial situation? |  | .534 |
| **Percentage variance explained = 62.94%** | 48.94 | 14.00 |
| **Kaiser-Meyer-Olkin (KMO)** | 0.849 | |
| **Bartlett’s test of sphericity** | p < 0.001 | |

IFDFW: InCharge Financial Distress/Financial Well-Being Scale; KMO: Kaiser-Meyer-Olkin

**Table S5: Factor analysis of the Household Food Insecurity Access Scale (Food-insecurity and eating-disorders in students, Lebanon, 2021-2022).**

| **Promax rotated matrix** | | |
| --- | --- | --- |
| **Factor** | **Factor 1** | **Factor 2** |
| HFIAS 4: In the past four weeks, did you or any household member have to eat some foods that you really did not want to eat because of a lack of resources to obtain other types of food? | .950 |  |
| HFIAS 6: In the past four weeks, did you or any other household member have to eat fewer meals in a day because there was not enough food? | .813 |  |
| HFIAS 3: In the past four weeks, did you or any household member have to eat a limited variety of foods due to a lack of resources? | .806 |  |
| HFIAS 2: In the past four weeks, were you or any household member not able to eat the kinds of foods you preferred because of a lack of resources? | .795 |  |
| HFIAS 1: In the past four weeks, did you worry that your household would not have enough food? | .748 |  |
| HFIAS 5: In the past four weeks, did you or any household member have to eat a smaller meal than you felt you needed because there was not enough food? | .525 |  |
| HFIAS 9: In the past four weeks, did you or any household member go a whole day and night without eating anything because there was not enough food? |  | .996 |
| HFIAS 7: In the past four weeks, was there ever no food to eat of any kind in your household because of lack of resources to get food? |  | .975 |
| HFIAS 8: In the past four weeks, did you or any household member go to sleep at night hungry because there was not enough food? |  | .450 |
| **Percentage variance explained = 70.21%** | 48.78 | 21.43 |
| **Kaiser-Meyer-Olkin (KMO)** | 0.446 | |
| **Bartlett’s test of sphericity** | p < 0.001 | |

HFIAS: Household Food Insecurity Access Scale; KMO: Kaiser-Meyer-Olkin

**Table S6: Factor analysis of the Pittsburgh Sleep Quality Index (PSQI) (Food-insecurity and eating-disorders in students, Lebanon, 2021-2022).**

| **Promax rotated matrix** | | |
| --- | --- | --- |
| **Factor** | **Factor 1** | **Factor 2** |
| Sleep disturbances | .762 |  |
| Sleepiness | .715 |  |
| Sleep latency | .648 |  |
| Sleep medication use | .557 |  |
| Subjective sleep quality | .498 |  |
| Habitual sleep efficiency |  | .838 |
| Sleep duration |  | .659 |
| **Percentage variance explained = 49.77%** | 32.99 | 16.77 |
| **Kaiser-Meyer-Olkin (KMO)** | 0.719 | |
| **Bartlett’s test of sphericity** | p < 0.001 | |

PSQI: Pittsburgh Sleep Quality Index; KMO: Kaiser-Meyer-Olkin

**Table S7: Factor analysis of the Eating Disorder Diagnostic Scale (Food-insecurity and eating-disorders in students, Lebanon, 2021-2022).**

| **Promax rotated matrix** | | |
| --- | --- | --- |
| **Factor** | **Factor 1** | **Factor 2** |
| EDDS_5: During the times when you ate an unusually large amount of food, did you experience a loss of control | .807 |  |
| EDDS_9: Eat large amounts of food when you didn’t feel physically hungry | .803 |  |
| EDDS_7: Eat much more rapidly than normal? | .792 |  |
| EDDS_8: Eat until you felt uncomfortably full? | .775 |  |
| EDDS_6: How many TIMES per month on average over the past 3 MONTHS have you eaten an unusually large amount of food and experienced a loss of control? | .759 |  |
| EDDS_4: During the past 3 months, have there been times when you have eaten what other people would regard as an unusually large amount of food (e.g., a pint of ice cream) given the circumstances | .730 |  |
| EDDS_12: If you have episodes of uncontrollable overeating, does it make you very upset? | .593 |  |
| EDDS_11: Feel disgusted with yourself, depressed, or very guilty after overeating? | .557 |  |
| EDDS_17: How many times per month on average over the past 3 months have you eaten after awakening from sleep or eaten an unusually large amount of food after your evening meal and felt distressed by the night eating? | .481 |  |
| EDDS_10: Eat alone because you were embarrassed by how much you were eating? | .477 |  |
| EDDS_13: Made yourself vomit? | .265 |  |
| EDDS_14: Used laxatives or diuretics? | .204 |  |
| EDDS_3 Body perception. Did your weight or figure affect how you judged yourself as a person? |  | .895 |
| EDDS_1 Body awareness: did you feel fat? |  | .889 |
| EDDS_2 Were you very afraid of gaining weight or becoming fat? |  | .835 |
| EDDS_18 How much do problems with food or with your body image affect your relationships with friends and family, your work performance, or your school performance? |  | .615 |
| EDDS_15 Fasted (skipped at least 2 meals in a row)? |  | .560 |
| EDDS_16 Engaged in more intense exercise specifically to counteract the effects of overeating? |  | .314 |
| **Percentage variance explained = 48.43%** | 37.75 | 10.68 |
| **Kaiser-Meyer-Olkin (KMO)** | 0.887 | |
| **Bartlett’s test of sphericity** | p < 0.001 | |

EDDS: Eating Disorder Diagnostic Scale; KMO: Kaiser-Meyer-Olkin

**Table S8: Factor analysis of the International Physical Activity Questionnaire (IPAQ)-Short Form per** **metabolic equivalent-minutes/ week (Food-insecurity and eating-disorders in students, Lebanon, 2021-2022).**

| **Promax rotated matrix** | |
| --- | --- |
| **Factor** | **Factor 1** |
| Moderate activities | .863 |
| Vigorous activities per day | .837 |
| Walking physical activity per day | .276 |
| **Percentage variance explained** | 50.71 |
| **Kaiser-Meyer-Olkin (KMO)** | 0.501 |
| **Bartlett’s test of sphericity** | p <0.001 |

IPAQ: International Physical Activity Questionnaire Short Form; KMO: Kaiser-Meyer-Olkin

**Table S9: Factor analysis of the Mediterranean Diet Adherence Screener (Food-insecurity and eating-disorders in students, Lebanon, 2021-2022).**

| **Promax rotated matrix** | |  |  |  |
| --- | --- | --- | --- | --- |
| **Factor** | **Factor 1** | **Factor 1** | **Factor 1** | **Factor 1** |
| MEDAS6: How many servings (12 g = 1Tablespon) of butter, margarine, or cream do you consume per week? | .775 |  |  |  |
| MEDAS5: How many servings of red meat, hamburger, or sausages do you consume per week? A full serving is 100–150 g. | .706 |  |  |  |
| MEDAS7: How many carbonated and/or sugar-sweetened beverages do you consume per week? | .647 |  |  |  |
| MEDAS10: How many servings of fish/seafood do you consume per week? (100–150 g of fish, 4–5 pieces or 200 g of seafood) |  | .629 |  |  |
| MEDAS8: Do you drink wine? How much do you consume per week? |  | .486 |  |  |
| MEDAS12: How many times do you consume nuts per week? (1 serving = 30 g) |  | .486 |  |  |
| MEDAS13: Do you prefer to eat chicken, turkey or rabbit instead of beef, pork, hamburgers, or sausages? |  | .481 |  |  |
| MEDAS4: How many pieces of fruit (including fresh-squeezed juice) do you consume per day? |  |  | .696 |  |
| MEDAS14: How many times per week do you consume boiled vegetables, pasta, rice, or other dishes with a sauce of tomato, garlic, onion, or leeks sautéed in olive oil? |  |  | .524 |  |
| MEDAS3: How many servings of vegetables do you consume per day? Count garnish and side servings as 1/2 point; a full serving is 200 g. |  |  | .497 |  |
| MEDAS1: Do you use olive oil as the principal source of fat for cooking? |  |  | .433 |  |
| MEDAS2: How much olive oil do you consume per day (including that used in frying, salads, meals eaten away from home, etc.)? |  |  | .265 |  |
| MEDAS9: How many servings (150 g) of pulses (grains) do you consume per week? |  |  |  | .590 |
| MEDAS11: How many times do you consume commercial (not homemade) pastry such as cookies or cake per week? |  |  |  | .528 |
| **Percentage variance explained = 43.31** | 14.15 | 11.47 | 9.55 | 8.13 |
| **Kaiser-Meyer-Olkin (KMO)** | 0.556 | | | |
| **Bartlett’s test of sphericity** | <0.001 | | | |

MEDAS: Mediterranean Diet Adherence Screener; KMO: Kaiser-Meyer-Olkin
